# Natural Metallic Nanoparticles for Application in Nano-Oncology

**DOI:** 10.3390/ijms21124412

**Published:** 2020-06-21

**Authors:** Edouard Alphandéry

**Affiliations:** 1 Institut de Minéralogie, de Physique des Matériaux et de Cosmochimie, Paris Sorbonne Université, Muséum National d’Histoire Naturelle, UMR CNRS 7590, IRD, IMPMC, 75005 Paris, France; edouardalphandery@hotmail.com; Tel.: +33-632-697-020; 2 Nanobacterie SARL, 36 boulevard Flandrin, 75116 Paris, France; 3 Institute of Anatomy, UZH University of Zurich, Winterthurerstrasse 190, CH-8057 Zurich, Switzerland

**Keywords:** natural metallic nanoparticle, natural nanoparticle, bio-synthesized nanoparticle, plant synthesized nanoparticle, bacterial nanoparticle, magnetosomes, cancer, alternating magnetic field, magnetic hyperthermia, glioblastoma, GBM, anti-tumor activity, nanomedicine, nanoparticle, magnetotactic bacteria

## Abstract

Here, the various types of naturally synthesized metallic nanoparticles, which are essentially composed of Ce, Ag, Au, Pt, Pd, Cu, Ni, Se, Fe, or their oxides, are presented, based on a literature analysis. The synthesis methods used to obtain them most often involve the reduction of metallic ions by biological materials or organisms, i.e., essentially plant extracts, yeasts, fungus, and bacteria. The anti-tumor activity of these nanoparticles has been demonstrated on different cancer lines. They rely on various mechanisms of action, such as heat, the release of chemotherapeutic drugs under a pH variation, nanoparticle excitation by radiation, or apoptotic tumor cell death. Among these natural metallic nanoparticles, one type, which consists of iron oxide nanoparticles produced by magnetotactic bacteria called magnetosomes, has been purified to remove endotoxins and abide by pharmacological regulations. It has been tested in vivo for anti-tumor efficacy. For that, purified and stabilized magnetosomes were injected in intracranial mouse glioblastoma tumors and repeatedly heated under the application of an alternating magnetic field, leading to the full disappearance of these tumors. As a whole, the results presented in the literature form a strong basis for pursuing the efforts towards the use of natural metallic nanoparticles for cancer treatment first pre-clinically and then clinically.

## 1. Introduction

Many studies have reported the potential of metallic nanoparticles (NP) of different compositions, e.g., Pt, Pd, Au, Fe, Ag, Ni, Cu, Se, or their oxides, for cancer treatments, through various mechanisms of action, such as the local production of heat [1], controlled drug release [2], or an enhancement of the effect of radiation [3]. While the most straightforward definition of a NP is that of a material with at least one dimension at the nanometer scale, it can be widened to include any compound that would cause or be responsible for a phenomenon at this scale, e.g., an enhanced catalytic activity [4]. Although this broader definition has a more sound scientific ground, it is rarely used, since the tools at our disposal to directly observe or measure nano-metric phenomena, specifically dynamic ones, are still lacking. In order for a cancer treatment to be optimal, it should on the one hand efficiently target cancer cells and, on the other hand, be controllable on demand. This approach should maximize its efficacy while minimizing its side effects. Using nanoparticles, it is possible to fulfill these two objectives. Indeed, specific cancer cell targeting can be achieved through: (i) passive targeting also designated as EPR effect where nanoparticles passively diffuse through the angiogenic blood vessels irrigating the tumor, (ii) active targeting by attaching a targeting ligand to the nanoparticles that specifically recognize a cancer cell receptor, or (iii) magnetic targeting by applying a magnetic field on nanoparticles that trigger nanoparticle diffusion towards the tumor [5]. Furthermore, in many interesting cases described in the literature, nanoparticles can be activated on demand, for example by being illuminated with a laser or by being exposed to an alternating magnetic field, X-rays, or ultrasounds [5]. Most of them report results that are obtained with nanoparticles that were chemically synthesized using methods, such as precipitation, sonication, ball milling, thermal decomposition, spray pyrolysis, thermal hydrolysis, and sol–gel [4,5]. These chemical syntheses suffer from drawbacks, such as the uses of toxic solvents/reagents, high temperature/pressure, or additives needing to be added for metallic NP stabilization during the reaction of metallic NP formation. Besides, the methods of nanoparticle chemical synthesis are not always reproducible, i.e., the same nanoparticle parameters, such as nanoparticle sizes, distributions in sizes, geometry, shapes, cannot always be obtained from one batch to another. Although this is rarely described in the literature, such variations can be due to the use of containers made of different materials or with different volumes or to local changes in temperatures or pressures at the nanometer scale that are not observable with standard sensors [6]. By naturally synthesizing metallic NP, toxic products can be avoided and the environment can be respected since biological substances that are necessary for such synthesis are either used in limited quantity, e.g., for plants, or can be grown/amplified almost indefinitely without requiring a lot of natural resources, as it is the case for bacteria. In addition, living organisms, such as magnetotactic bacteria, may minimize variations in NP properties by using their own metabolism to produce NP, which might, to some extent, prevent the impact of environmental changes on NP properties [7].

After a presentation of the different types of natural metallic NP that have been described in the literature, I summarize the various ways in which they can be obtained, which depend on the type of biological material used for the synthesis, i.e., essentially fungus, yeast, plant extracts, and bacteria. The mechanisms of action that were associated with metallic NP anti-tumor activity were reported to involve, among others, apoptosis and anti-angiogenesis and depend on NP composition, the association (or not) of metallic NP with a chemotherapeutic drug, or the activation of NP through the application of radiation. Whereas anti-tumor activity of the majority of natural metallic NP was assessed in vitro, in vivo anti-tumor efficacy was also demonstrated with magnetosomes, which are iron oxide nanoparticles that are synthesized by magnetotactic bacteria. Indeed, it was shown that the administration of magnetosomes inside intracranial GBM mouse tumors followed by the repetitive heating of these tumors under alternating magnetic field application leads to full tumor disappearance. 

## 2. Advantages of Natural Chemical Synthesis of Metallic Nanoparticles

Naturally synthetizing metallic nanoparticles (NP) presents a number of advantages, such as: (i) the possibility to mass produce *metallic* NP without using toxic compounds [6], (ii) the ease with which the raw material can be found, (iii) a secured working environment due to the use of non-toxic biological materials, (iv) a reasonable cost in most cases, (v) a respect of the environment, (vi) the existence of a large number of biological compounds that can be chosen for the synthesis, such as plant metabolites acidic compounds, aldehydes, alkaloids, amino acids, alkaloids, aromatic amines, flavonoids phenolic compounds, ketones, phenols, phyto-proteins, polysaccharides, proteins, saponins, steroids, sugar compounds, tannins, terpenoids, vitamins, fungus, yeast, bacteria, virus, and bio-polymer [8], (vii) biological entities, which can have multiple functions at the same time, often combining a reducing capacity of metallic ions with either a stabilizing role, e.g., for starch, dextran, alginate, cellulose, chitin, yeast/fungus extracts, or a chemotherapeutic effect, e.g., for curcumin [9,10,11], (viii) the possibility to reach optimized NP properties, such as a high NP production yield, or a desired NP size/shape, by adjusting various synthesis parameters, such as the type of biological and metallic precursor materials used, the ratio between the quantities of precursor and biological material, the pH, temperature, or time of the reduction reaction [12], and, finally, (ix) the possibility to genetically modify certain living organisms to optimize metallic ion bio-mineralization by these organisms [11].

## 3. The Different Types of Bio-Synthesized Metallic Nanoparticles

The various metallic bio-synthesized NP were shown to be of various compositions. First, CeO_2_ NP, which have excellent catalytic properties due to their large bang gap and exciton binding energies, can be obtained by mixing Ce precursors, such as cerium acetate, cerium nitrate, and cerium chloride, with plant extracts acting as reducing agents, leading to CeO_2_ crystalline spherical NP of 4–19 nm [13]. Second, Ag NP, which have been shown to display anti-tumor and anti-bacterial activities, can be produced through the reaction between silver nitrate salt (AgNO_3_) and different reducing agents, such as plant extracts, i.e., originating from proteins, carbohydrates, flavonoids, phenols, vitamins, capsaicinoids, curcumin, basil, Pelargonium graveolens, Podophyllum hexandrum, Cinnamomum camphora, bark, callus, flower, fruit, stem, seed, peel, leaves, petals, and rhizome, to yield stable crystalline spherical/triangular Ag NP of a wide range of different sizes of 5–80 nm, depending on the synthesis method [9,14,15]. Third, Au NP, which have been shown to induce apoptotic cancer cell death, can be produced by mixing chloroauric acid (HAuCl_4_) with plants, plant extracts, or leafs, such as Brassica Junceae, Medicago Sativa, Picea Mariana, curcumin, quercetin, lemon grass, Neem, and Pear fruit, typically resulting in triangular, pentagonal, hexagonal, and spherical Au NP of 2 to 300 nm [9,10,14]. Fourth, Cu/CuO NP, which can be obtained cost effectively while using plant extract reducing agents, such as L-ascorbic acids, amino acids, reducing sugars, and phenolic compounds, typically consist of spherical Cu/CuO NP of 3 to 250 nm [15]. Fifth, Se NP can be produced through the reduction of a selenium precursor, such as SeO_3_^2^^−^ by leaf extracts of Capsicum Annuum, fruit extracts of Vitis Vinifera, polysaccharides extracted from Undaria Pinnatifida, typically resulting in spherical Se NP of 3–18 nm [16,17,18]. Sixth, Ni/NiO NP can be synthesized through the reduction of Nickel ion sources, such as Ni(NO_3_)_2_ or NiCl_2_, by: (i) different parts of plants, such as leaves, petals, and roots, e.g., of Medicago, Ocimum sanctum, Hibiscus rosa-sinensis, and rambutan peel waste, (ii) products, such as C_2_H_4_, resulting from the pyrolysis of petals, (iii) fungus, such as Aspergillus aculeatus, or (iv) Nickel resistant bacteria MRS-1, typically yielding well-dispersed stable face-centered cubic Ni NP of 1–200 nm [19]. Seventh, Pd NP, which can be obtained through the reduction at room temperatures of a Pd source, such as PdCl_2_ by plant or leaf extracts, e.g., of cinnamomum camphora, coffee, tea extract, or plant polymer gum ghatti, typically result in cubic faced-centered crystallized NP of 2–60 nm [9]. Eight, the formation of Pt NP of 2–23 nm can result from the reaction at 95 °C between a Pt solution such as chloroplatinic acid hexaydrate (H_2_PtCl_6_·6H_2_O) and various plant extracts, e.g., of Diopyros kaki, Cacumen platycladi extract, and Terminalia chebula [9]. Nineth, TiO_2_ NP can be obtained through the reduction of Titanium dioxide hydrate by some plant extracts [15]. Tenth, hexagonal, spherical, rod, and crystalline ZnO NP of 9–180 nm can be produced through the reduction of different zinc compounds, e.g., zinc acetate, zinc nitrate, and zinc sulfate, by: (i) various plants, stem, leaf, flower, fruit, trees, herbs, ornamentals, and seaweeds, Aloe vera, palm pollen, and dried leaves, which accumulate zinc [15]. Eleventh, the synthesis of different types of iron-based NP was also reported, which could result from the reduction of iron sources by plant extracts (Amaranthus spinosus leaf aqueous extracts) or various bacteria intracellularly or extracellularly [15].

## 4. The Various Bio-Synthesis Methods of Metallic Nanoparticles

First, NP synthesis can be carried out using whole plants or plant extracts, originating, for example, from stem, leaf, flower, fruit, root, latex, seed, and seed coat, which are mixed or brought into contact with a solution of metal salt during various lengths of time at various temperatures and pH under agitation (or not). This leads to the reduction of metallic ions into NP. Furthermore, certain plants, called hyper-accumulator, which are accumulating high metal concentrations, can be used for improving this fabrication process [13]. Although plant extracts appear to be very appealing for synthesizing metallic NP, there was not any attempt to the author knowledge to purify these extracts in order to make them compatible with pharmaceutical regulation and, hence, be able to use them in a pharmaceutical production process. 

Second, the production of metallic NP can also be achieved by fungus or yeasts, especially those that absorb large quantities of metals from their environment [20], often involving extracellular proteins/enzymes and/or mechanisms of bio-precipitation, chelation, or intra-cellular NP crystallization. Examples of yeast and fungus that can form NP include a marine strain of ascomycetous yeast Yarrowia Lipolytica that produce Ag NP [21], fungi Cladosporium Oxysporum that convert Au ions into Au NP [22], and Candida Utilis that bio-synthesize Ag NP [23]. 

Third, metallic NP can be produced by marine algae, which comprise biological compounds and secondary metabolites that can synthesize metallic NP [24] Examples of such algae include Codium capitatum for the Ag NP biosynthesis [24], extracts of Chlorella vulgaris for Pd NP production [21], extracts of Turbinaria Conoides for Au NP obtention [24], and Sargassum Muticum extracts to yield ZnO NP [24]. 

Fourth, metallic NP can be formed by viruses using proteins of their capsids that, can under certain circumstances, interact with metallic ions and reduce them into NP [20], e.g., enabling Pd NP synthesis [25] or Zn/Cd sulfide NP synthesis by M13 bacteriophage [26]. 

Fifth, it is also possible to use certain nutrients or natural materials, such as egg white proteins and honey for metallic NP production, e.g., CeO_2_ NP [13]. Mechanisms of such synthesis were reported to rely on interactions between Ce^3+^ and oppositely charged egg white proteins or hydroxyl/amine groups of carbohydrates, enzymes and vitamins, of honey matrix [13]. 

Sixth, it was also reported that metallic NP could be produced by gram-positive or gram negative bacteria, intracellularly or extracellularly. The most commonly reported metallic NP of this type are composed of iron oxide, possibly due to the high level of bio-compatibility that is associated with such composition, multiple potential uses of iron by bacteria, and ubiquitous iron presence in the environment [27]. Such bacterial synthesis involves certain proteins/enzymes, such as dodecameric (Dps), ferritin, and encapsulin, which participate in oxydo/reduction reactions, leading to NP formation. Magnetotactic bacteria, such as MSR-1 and AMB-1, are responsible for the intracellular fabrication of membrane bounded Fe_3_O_4_ NP, called magnetosomes [28], while iron-reducing bacteria, such as Geobacter Metallireducens or Actinobacter sp., produce extracellularly maghemite NP [27]. Both types of intracellular/extracellular synthesis occur in the presence of an iron source under different conditions of oxygenation. Other types of metallic NP can result from the reduction of various metallic ions different from iron, i.e.,: (i) selenite can be transformed into Se NP by Actinomycetes, Thauera selenatis, or E. coli [29,30,31,32,33,34,35], and (ii) Au chloride can be nano-formulated by the external cell membrane of Bacillus Subtilis [36]. In some cases, such synthesis involves specific enzymes, which favor electron transfers and oxydo-reduction reactions, such as NADH reductase, resulting for Pseudomonas aeruginosa bacterium in the intracellular production of Pd, Ag, Rh, Ni, Fe, Co, Pt, and Li NP [37], and leading for bacterium Streptomyces sp. LK-3 to the extracellular formation of Ag NP [38]. Concerning metallic NP synthesis outside of bacteria, it could be achieved with the help of extracellular polymeric substances (EPS), e.g., those of Alteromonas Macleodii that resulted in the fabrication of Ag NP [39]. Other types of metallic NP, such as those made of Te and Se, were also synthesized by certain bacterial species, such as Ochrobactrum sp. [40]. Metallic NP synthesis by bacteria does not only depend on the types of bacteria involved, but also on environmental factors, such as temperature, e.g., the synthesis of Ag NP by Morganella Psychrotolerans yielded triangular and hexagonal nanoplates at 20 °C and spherical nanoparticles at 25 °C. 

Finally, certain bio-polymers or bio-molecules possessing hydroxyl groups, such as agarose, glucose, sucrose, starch, and PEG, were used to synthesize metallic NP, e.g., CeO_2_ and Ni NP [13,19].

Table 1 summarizes the physico-chemical properties of the various natural metallic nanoparticles (metallic composition, organic part, size, shape, crystallinity, zeta potential, and presence of surface plasmon resonance peak, synthesis method).

## 5. In Vitro Anti-Tumor Activities of Bio-Synthesized Metallic Nanoparticles

The anti-tumor activity of metallic NP composed of Ni/NiO_2_ [64,65], ZnO [66], Ag [67], Au [68,69,70], iron oxide [71], and Se [35] has been demonstrated on liver, colon, breast, skin, and hepatoma cancer cells [35,65,66,67,68,69,70,71,72], highlighting the potential of these bio-synthesized metallic NP for the treatment of various cancers. When natural metallic NP were incubated with cancer cells, different levels of cytotoxicity were observed, depending on NP/cancer cell type, i.e., the percentage of living cells decreased: (i) from 80% for 87 µg/mL of ZnO NP incubated with HePG2 during 24 h to 20% for 2800 µg/mL of ZnO NP incubated with these cells [67], (ii) from 90% for 0.9 µg/mL of Ag NP incubated with MCF-7 breast cancer cells during 24 h to 10% for 30 µg/mL of Ag NP incubated with these cells [68]. A comparison between the efficacy of metallic NP of various compositions was carried out for Au and Ag NP synthesized by plants Plumbago zeylanica, Commelina nudiflora, and Cassia auriculata, which indicated that Ag was more cytotoxic than Au towards various cancer cell lines [72]. More comparative studies deserve to be carried out in order to determine which metallic NP composition would potentially yield the most efficient anti-tumor effect. Three cases can be distinguished concerning the impact of NP composition on anti-tumor activity. First, the biological material used for metallic NP synthesis is cytotoxic and it ends up at the surface or in the composition of metallic NP, inducing a cytotoxicity towards cancer cells, which is larger than that of the free biological material, as observed for Ni NP associated with Verbascoside [65], or Au NP combined with Cur [70,73]. Second, a cytotoxic extract of plant can be added to metallic NP after nanoparticle synthesis; this fabrication process presented the advantage of offering the choice between different types of cytotoxic compounds that can be attached to the metallic NP backbone. Thirdly, bio-synthesized metallic NP may not comprise any natural cytotoxic compounds, in which case cytotoxicity might be induced by the metallic NP backbone or by other mechanisms of tumor destruction than purely cytotoxic ones, such as those involving the immune system. In vitro studies of tumor cell destruction have highlighted a whole variety of different types of mechanisms of tumor cell destruction, such as anti-angiogenesis, as observed for liver cancer cells brought into contact with ZnO NP synthesized by Sargassum muticum extracts [67], apoptosis induced by the expression of pro-apoptotic Bax protein in MCF-7 human breast cancer cell line in the presence of Au NP [73], pH-controlled drug release, e.g., Gingerol 6G or Cur could be released from IONP in this manner [72], the delivery of the two chemotherapeutic drugs doxorubicin and Quercetin associated with Au NP by infrared light illumination [74], mechanical damage/disruption of cell membrane by CeO NP [75,76], DNA damage that is induced by Au and Ag NP [77,78], the prevention of drug aggregation via drug conjugation to Se NP that enhances drug bio-availability [79], and tumor cell targeting of drug-NP conjugates through the specific interactions between drug compounds (carbohydrates) and cell receptors (lectins) [80]. 

## 6. Preparation and In Vivo Anti-Tumor Assessment of Magnetosomes 

Only few bio-synthesized metallic NP had their anti-tumor efficacy examined in vivo. The reason for that may be that despite their potential, many metallic NP formulations may not have reached a sufficiently high level of accomplishment or be fully compatible with pharmaceutical regulation to warrant in vivo evaluations. Even so, the presence of biological material, such as plant extracts in the final formulation, is often perceived as an advantage due to the natural origin of such substance, it might be difficult to obtain it reproducibly and identically from batch to batch. A detailed description of the methods used for purifying metallic NP, as well as the exact metallic NP composition resulting from such methods, are also often lacking. Furthermore, metallic NP characterization should go beyond the sole estimate of nanoparticle size, which is often the only reported NP property.

As a first example, Au NP associated with Resveratrol (RES–Au NP) were administered to mice bearing liver tumors, yielding better anti-hepatoma efficacy than for free Resveratrol, [81].

**Figure 1:**
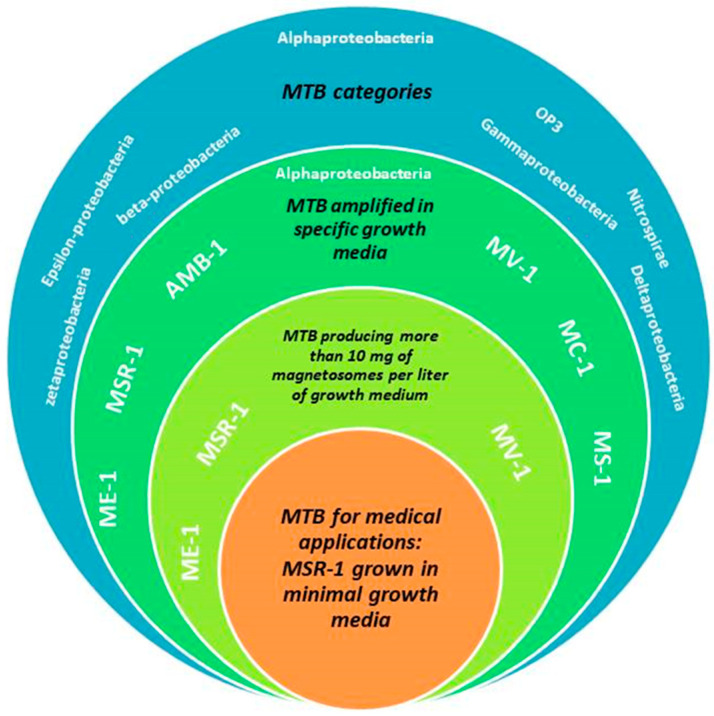
A schematic diagram presenting the various categories of magnetotactic bacteria (blue background), species of magnetotactic bacteria belonging to Alphaproteobacteria that can be amplified in specific growth media (dark apple green background), species of magnetotactic bacteria belonging to Alphaproteobacteria that can be amplified in specific growth media and can produce more than 10 mg of magnetosomes per liter of growth medium (yellow green background), MSR-1 specie belonging to Alphaproteobacteria that can be amplified in specific growth media, can produce more than 10 mg of magnetosomes per liter of growth medium, and can be amplified in minimal growth media not containing peptone, yeast extract, toxic CMR products, and heavy metals.

As a second example, magnetosomes, which are iron oxide nano-minerals surrounded by biological material made of lipids/proteins [82], synthesized by magnetotactic bacteria (MTB), have been developed methodically, in order to enable their administration to a living organism. First, the MTB species, which is the best suited for medical applications, was determined. MTB are ubiquitous in the environment, belonging to a broad range of different classes, as shown in Figure 1, i.e., zetaproteobacteria, beta-proteobacteria, Gammaproteobacteria, Deltaproteobacteria, Epsilon-proteobacteria, Nitrospirae, OP3, and Alphaproteobacteria. However, only certain species of Alphaproteobacteria such as Magnetospirillum gryphiswaldense MSR-1, Magnetospirillum magneticum AMB-1, Magnetovibrio MV-1, Magnetococcus sp. MC-1, Magnetospirillum magnetotacticum MS-1, and Magnetospirillum sp. ME-1, could be grown under controlled conditions [83]. Among these species, MSR-1 seems particularly interesting. Indeed, it was shown that MSR-1 could be amplified with a good yield, i.e., the production of 10 mg of magnetosomes per liter of growth medium, by using a reduced growth medium that is devoid of chemicals of animal/cell origin, such as peptone and yeast extract, which are often used to promote bacterial growth, and of toxic substances, such as CMR (carcinogenic, mutagenic, reprotoxic) products and heavy metals. It yielded pure iron oxide nanoparticles, using a production method that abides by pharmaceutical production standards [7]. 

**Figure 2:**
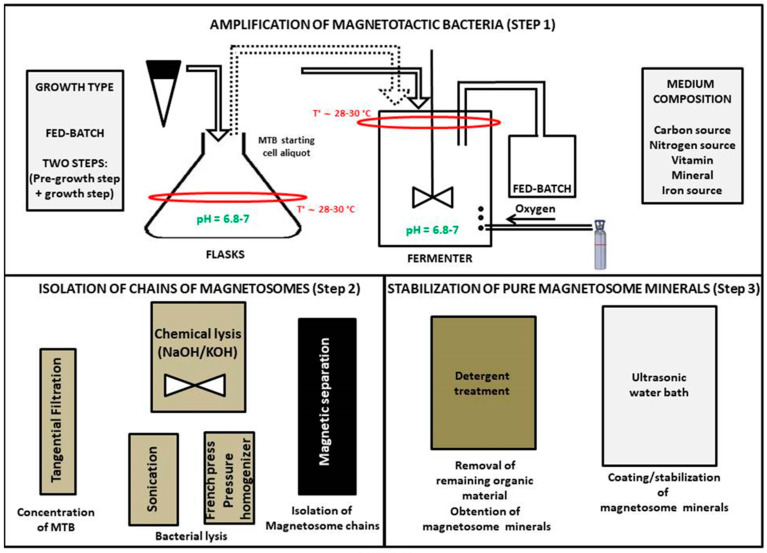
Schematic diagrams presenting the different steps of magnetosome production, which include: step 1) growth/amplification of magnetotactic bacteria, this step being divided between a pre-growth step where magnetotactic bacteria (MTB) are amplified without a source of iron and a growth step during which MTB are grown by being fed with a fed-batch medium containing a source of iron under sustained oxygen gas bubbling to promote magnetosome production, step 2) extraction of magnetosomes from MTB by mixing MTB with KOH and isolating extracted magnetosomes with a magnet, step 3) a treatment with detergents or heat to obtain magnetosome minerals and a sonication of these minerals with a synthetic coating (Poly-L-Lysine) to yield stable coated magnetosome minerals (M-PLL).

Magnetotactic bacteria being gram negative, they contain LPS at the surface of their membrane, which should be removed for injection, notably to prevent skeptic shock. For that, an extraction/purification method was developed, in which MTB and extracted magnetosomes were treated with detergents (KOH or NaOH) and heat in order to remove biological material, including LPS from the magnetosome minerals. Finally, the minerals thus obtained were coated with several synthetic substances, such as Poly-L-lysine under sonicating conditions, in order to yield nano-formulated magnetosome minerals coated with Poly-L-lysine (M-PLL), which were stable and non-pyrogenic [84]. Figure 2 presents a schematic diagram with the different steps of the production of M-PLL, which include: (i) growth and amplification of MSR-1 magnetotactic bacteria starting with a pre-amplification of MTB without feeding the bacteria with iron followed by growth of MTB under iron fed-batch conditions (step 1), (ii) the extraction of magnetosomes from MTB, and (iii) the purification of magnetosomes to remove organic materials and yield magnetosome minerals and finally coating of magnetosome minerals with poly-L-lysine (step 3). 

**Figure 3:**
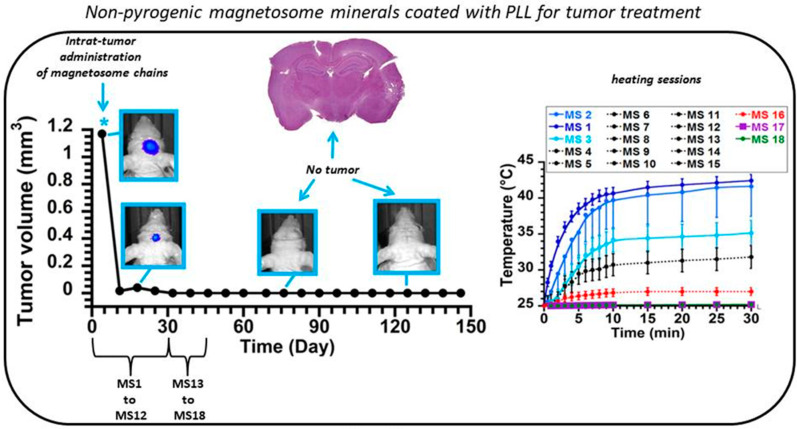
Treatment of a mouse bearing an intracranial GBM tumor of 1.5 mm^3^, which receives at the center of its tumor 500–700 µg of M-PLL, followed by 27 applications of an alternating magnetic field of 27 mT and 200 kHz, each session lasting for 30 min, and resulting in moderate temperature increase of 0 to 18 °C. The decrease of tumor volume down to 0 mm^3^ 10 days following the beginning of the treatment as well as the absence of tumor in histological analysis of treated tumor brain slides collected 75 days and 125 days following the beginning of the treatments reveal the full tumor disappearance induced by the treatment. “reproduced with permission in a slightly different format from Biomaterials, V. 262, P. 259-272 (2017)”.

M-PLL were administered at a concentration of 350–450 μg in iron per mm^3^ of tumor at the center of GBM tumors of 1.5 mm^3^ grown in mouse brains to evaluate the anti-tumor efficacy of these nanoparticles. Mice were then exposed to 27 magnetic sessions (MS), during which an alternating magnetic field of 200 kHz and 27 mT was applied during 30 min. [84]. For a typical mouse, Figure 3 shows the rapid tumor disappearance resulting from the treatment, which is highlighted by the decrease of tumor volume, as deduced from bioluminescence measurements and the absence of tumor cells observed through histological analysis of treated mouse brains 75 and 125 days following the beginning of the treatment [84]. The tumor temperature recorded during each session increased moderately, not exceeding 43 °C, indicating that such treatment might be compatible with the treatment of a human brain, which might withstand a moderate temperature increase. 

**Figure 4:**
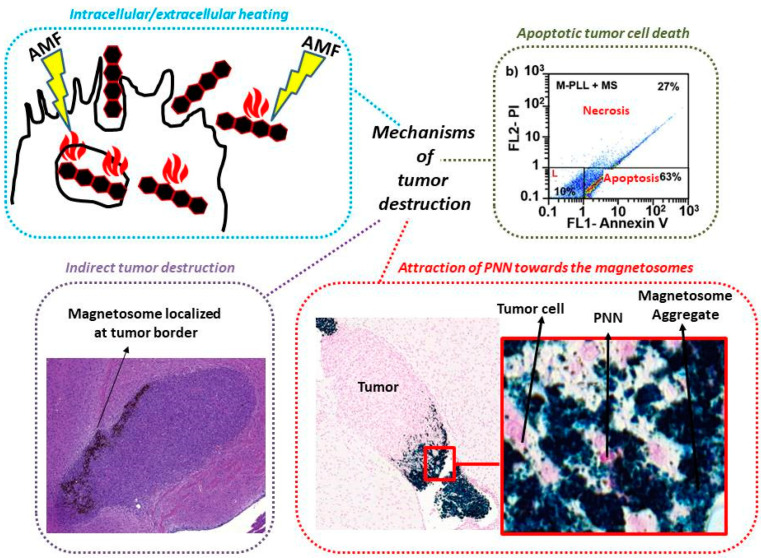
Schematic diagram presenting the possible mechanism of actions associated with anti-tumor activity, i.e.,: (i) localized heat produced at magnetosome location, either inside or outside cells depending on whether magnetosomes internalize in tumor cells or remain localized outside these cells, (ii) an apoptotic mechanism as highlighted when magnetosomes were brought into contact with U87-Luc cells and exposed to one magnetic session, (iii) mechanism of indirect tumor destruction involving magnetosomes covering the border of GBM tumor, and (iv) the attraction of immune cells (PNN) by the magnetosomes since PNN were observed near the magnetosomes in GBM tumors following a magnetic session although a direct link between the presence of PNN and the anti-tumor activity was not established.

Figure 4 illustrates, through a series of fours schematic diagrams, the different mechanisms that could be involved in the anti-tumor activity, i.e.,: (i) localized heat induced at magnetosome location that could occur inside or outside tumor cells, depending on whether (or not) magnetosomes have internalized in tumor cells [54,85], (ii) a mechanism of tumor cell death dominated by apoptosis as was observed during in vitro studies where magnetosomes were brought into contact with GBM cells and then exposed to one magnetic session [53,84], (iii) anti-tumor activity due to the presence of the magnetosomes within tumor margin, (iv) the attraction of certain immune cells, such as PNN, in the region where magnetosomes are located, even so a direct link between the anti-tumor activity and the presence of PNN has not yet been established [53,84]. In cases where magnetosomes are heated under the application of radiation, e.g. a laser or an alternating magnetic field, heat appears to be the dominant mechanism of action since tumor disappearance is not observed in the absence of heat, and magnetosomes are therefore classifiable as medical devices.

Table 2 summarizes the in vitro and in vivo anti-tumor efficacies obtained with the various types of nanoparticles presented in Table 1.

## 7. Conclusions

The development of metallic nanoparticles for cancer treatment is rapidly expanding. This is due to the wide range of mechanisms of action that such a system can trigger, which depends on the type of metallic NP used. It includes controlled chemotherapeutic drug delivery, localized heating, and amplification of radiation. Most efforts in this field have focused on chemical synthesis to obtain metallic NP. However, such synthesis suffers from a number of drawbacks, notably the use of toxic products that should be avoided for medical applications. This is the reason why it has recently been suggested to follow natural synthesis methods, using plant extracts, fungus, yeast, or bacteria, to produce metallic NP. Such production usually relies on the reduction of metal ions by biological material, which results in the crystallization of metallic ions into metallic NP. The anti-tumor activity of most metallic NP has been demonstrated in vitro, where some of the underlying mechanisms have been described as involving anti-angiogenesis or tumor cell apoptosis, DNA damage, or membrane disruption. Following a specific purification method that enables the removal of endotoxins and immunogenic biologic material from nanoparticles, metallic NP, called magnetosomes, which are iron oxide nanoparticles that are synthesized by magnetotactic bacteria, have been administered inside glioblastoma tumors grown inside mouse brains, and further excited by several applications of an alternating magnetic field, resulting in several heating sessions that led to full tumor disappearance. Therefore, it has been demonstrated that natural metallic nanoparticles have the potential to destroy tumors pre-clinically. The next step will consist in assessing the efficacy and safety of these natural metallic NP clinically. 

## Figures and Tables

**Table 1:** Properties of different types of natural metallic nanoparticles, including the metallic composition, the organic part, the size, the shape, the crystallinity, the zeta potential, the presence (or not) of impurities, and the synthesis method.

**Metal. Comp.** **(NP, NW)**	**Organic Part** **(Capping/Stabilizing Agent)**	**Size (nm)** **Shape** **Crystallinity** **Zeta Potential (ZP in mV)**	**Surface Plasmon Resonance**	**Impurities**	**Synthesis**	**References**
NiO NP	Plant extract	21 nm Sphere Crystalline ZP = −10 mV	NA	NA	Plant extract of *Geranium wallichianum* acting as reducing and capping agent	[41], Abbasi 2019
Pt NP	Extract of dates	1–3 nm Sphere Crystalline	321 nm, 329 nm.	K, Na, Mg, Ca, P, Fe, Cu, Zn, Cd, Mn	Purified date extract mixed with H2PtCl6 at different pH and temperatures	[42], Al-Radadi 2019
Pd NP	Bis-phthalate or plant metabiltes	10–50 nm Sphere Crystalline ZP = −23 mV	460 nm	NA	Extract of *Moringa oleifera* flower react with Pd(II) Species	[43], Anand 2016
Pd NP	phenols and flavonoids	6–18 nm Sphere Crystalline	NA	NA	PdCl_2_ solution was mixed with aqueous white tea extract at 40 °C.	[44], Azizi 2017
Au NP	Alkanoids/flavonoids	10–42 nm Pentagone + triangular ZP = 42 m V	500–600 nm	NA	extracts of Zataria multiflora leaves mixed with chloroauric acid (HAuCl_4_) aqueous solution	[45], Baharara 2016
ZnO NP	leaf extract	29 nm triangle, radial, hexagonal, rod, and rectangle Crystalline	NA	NA	zinc nitrate mixed with leaf extract of Eclipta prostrata dueing 48 h	[46], Chung 2015
Cu NP	Biomolecules of leaf extract	23–57 nm spherical, hexagonal and cubical Polycrystalline	565 nm	NA	copper acetate Cu(OAc)_2_ mixed with aqueous extract of *E. prostrata* for 24 h.	[47], Chung 2017
Te NW	NA	Length (a few µm), diameter = 26 nm, Wire	NA	NA	Telluric acid mixed with starch and heated at 160 °C for 15 h.	[48], Crua 2019
Se NP	hawthorn fruit extract	113 nm	NA	NA	Sodium selenite was mixed with extracts of hawthorn fruit under stirring for 12 h.	[49], Cui 2018
Ag NP	bioactive molecules of plant extracts	98 nm Sphere ZP = −32 mV	434 nm	NA	Silver nitrate mixed with Cynara scolymus extract in ultrasonic bath for 30 min.	[50], Erdogan 2019
CeO_2_ NP	Protein of fresh egg white	8-17 nm Sphere Crystalline	NA	NA	Ce(NO_3_)_3_.6H_2_O mixed with fresh eggs at 60 °C.	[51], Kargar 2015
**Metal. Comp.** **(NP, NW, QD)**	**Organic Part** **(Capping/Stabilizing Agent)**	**Size (nm)** **Shape** **Crystallinity** **Zeta Potential (ZP, mV)**	**Surface Plasmon Resonance**	**Impurities**	**Synthesis**	**Ref**
Fe NP	Secondary metabolites	100 nm Round	NA	NA	Rosemary plant extracts mixed with FeSO4.	[52], Farshchi 2018
Fe_2_O_3_NP Yield: 10 mg per liter of growth medium	Iron oxide mineral coated by bacterial lipids/proteins	40 nm (average) Cubo-octahedric Chain arrangement Crystalline ZP = −18 mV (pH 6)	NA	Other metals than iron (% of iron relatively to other metals > 90%)	NP produced by MSR-1 *Gryphiswaldense* magnetotactic bacteria NP extracted as chains of magnetosomes from these bacteria. NP are pyrogenic	[53], Alphandéry 2017
Fe_2_O_3_ NP Yield: 10 mg per liter of growth medium	Iron oxide mineral part coated by synthetic coating (CA, CMD, OA, PEI, PLL, CHI, NER)	40 nm (average) Cubo-octahedric Chain arrangement Crystalline Zeta potential depends of coating	NA	Other metals than iron (% of iron relatively to other metals > 90%)	NP produced by MSR-1 *Gryphiswaldense* magnetotactic bacteria NP extracted as chains of magnetosomes from these bacteria, purification of chains to remove all organic material surrounding magnetosome mineral core, Coating of magnetosome core with various coating agents (CA, CMD, OA, PEI, PLL), CHI, NERI. Magnetosomes are non-pyrogenic	[54], Chalani 2017, 63, Mandawala 2017
Au NP	cocoa extract	150–200 nm Sphere Crystalline ZP = −50 mV	535 nm	NA	cocoa extract powder solution (reducing agent) mixed with HAuCl_4_	[55], Fazal 2014
Tb_2_O_3_ NP	NA	10 nm Crystalline ZP = −17 mV	NA	NA	Incubation of fungus *Fusarium oxysporumin* with Tb_4_O_7_.	[56], Iram 2016
MgO NP	Biomolecules	12–24 nm	215 nm	NA	Mixture of magnesium nitrate (MgNO_3_) with the aqueous extract of Penicillium.	[57], Majeed 2018
BaCO_3_ NP	NA	18 nm Crystalline Spherical + triangular	NA	NA	BaCl_2_ and Na_2_CO_3_ mixed with Mangifera seed extract at 120 °C for 6 h.	[58], Nagajyothi 2016
Bi_2_S_3_ NP	BSA	60 nm Crystalline Sphere ZP = −33 mV	NA	NA	Bi(NO_3_)_3_ and HNO_3_ added into bovine serum albumin solution.	[59], Nosrati 2019
Th NP	NA	100–1000 nm Crystalline	5 KeV	Au, Pd	Pineapple juice added to thorium nitrate.	[60], Pol 2018
CdS QD	Organic material	2–5 nm Crystalline	NA	NA	Two steps: 1/CdSO_4_ added to *C. sinensis* extracts for 3 days + 2/Na_2_S added for 4 days.	[61], Shivaji 2018
CoPt NP	Polyphenol	10 nm	NA	NA	co-reduction of cobalt (II) chloride and potassium tetrachloroplatinate (II) in the presence of polyphenols by using NaBH_4_ as a reduction agent	[62], Song 2016
MnO_2_ NP	NA	10–50 nm	300–400 nm	NA	Human serum albumin mixed with with manganese chloride in the presence of drug + photosensitizer	[63], Chen 2016

NP: Nanoparticle; NW: Nanowire.

**Table 2:** Summarizes the in vitro and in vivo anti-tumor efficacies of the various nanomaterials presented in Table 1.

Efficacy Results (In Vitro & In Vivo) of the Different Nanomaterials Presented in Table 1	References
cytotoxicity of NiO NP against HepG_2_ cancer cells (IC_50_ = 38 μg/mL)	[41], Abbasi 2019
Cytoxicity of Pt NP against MCF-7, HCT-116, HepG-2 cells (90 < IC_50_ < 290 µg/mL)	[42], Al-Radad i2019
Cytotoxicity of Pd NP towards A549 lung cancer cells No cytoxicity towards healthy peripheral lymphocytes	[43], Anand 2016
Pd NP have larger cytotoxicity toward human leukemia (MOLT-4) cells (IC_50_ = 0.006 μM) than tea extract (IC_50_ = 0.9 μM), doxorubicin (IC_50_ = 2 μM), or cisplatin (IC_50_ = 0.013 μM). NP relatively not cytotoxic towards healthy human fibroblast (HDF-a) cells. Cytotoxicity due to apoptosis/G2/M cell-cycle arrest NP have anti-oxydant activity	[44], Azizi 2017
Au NP anticancer activity against HeLa cells (through apoptosis)	[45], Baharara 2016
Cytotoxicity of ZnO NP towards Hep-G2 cells (ROS induce damage to DNA of the cells)	[46], Chung 2015
Cu NP are cytotoxic towards MCF-7 breast cancer cells	[47], Chung 2017
Te NW (concentration between 5 and 100 μg/mL) improves healthy cell proliferation/decreases cancer cell growth. Higher efficacy compared with chemical Te NW.	[48], Crua 2019
Se NP cytotoxic towards HepG_2_ cells (IC50 = 19 µg/mL) NP produce ROS Cellular death through apoptosis	[49], Cui 2018
Cytotoxicity towards MCF-7 breast cancer cells of Ag NP (10 μg/mL) and PDT (0.5 mJ/cm^2^) Due to mitochondrial damage and intracellular ROS production	[50], Erdogan 2019
No CeO_2_ NP cytotoxicitys on periodental fibroblast cells.	[51], Kargar 2015
Rosemary-FeNPs more cytotoxic towards 4T1 and C26 cancer cells than free Rosemary extract	[52], Farschchi 2018
Injection of magnetosomes in glioblastoma followed by several AMF applications leads to full tumor disappearance	[53], Alphandéry 2017
Magnetosome cytotoxicity towards GBM RG-2 and GL-261 cells under the application of an AMF of 200 kHz and 40 mT.	[54], Hamdous 2017, 63, Mandawala 2017
Au NP cytotoxic towards epidermoid carcinoma A431 cells upon laser irradiation laser at 800 nm (6 W/cm^2^).	[55], Fazal 2014
Tb_2_O_3_ NP cytotoxic towards MG-63 and Saos-2 osteosarcoma cancer cells (IC_50_ = 0.102 μg/mL) Tb_2_0_3_ NP not cytotoxic towards primary osteoblasts up to 0.373 μg/mL.	[56], Iram 2016
Cytotoxicity of MgO NP towards A-549 human lung cancer cells (IC_50_ = 100 μg ml^−1^ after 24 h incubation) Less cytotoxicity of MgO NP twoards healthy vero cells (IC_50_ = 140 µg/mL)	[57], Majeed 2018
BaCO_3_ NP cytotoxic towards cervical carcinoma cells	[58], Nagajyothi 2016
Possibility to add Curcumin at the surface of bismuth sulfide NP and to induce cytotoxicity towards HT-29 cells by release of CUR.	[59], Nosrati 2019
Th NP cytotoxic towards A 375 melanoma cells.	[60], Pol 2018
CdS QD cytotoxic towards A549 lung cancer cells.	[61], Shivaji 2018
CoPt have better biocompatibility/lower toxicity than previously reported Co NP, Co@Au NP, and CoPt NP. → due to good biocompatibility/anti-oxidation potential of polyphenols that prevent cobalt release.	[62], Song 2016
MnO_2_ NP cytotoxic towards 4T1 breast cancer cells	[63], Chen 2016

## Abbreviations

AMF	Alternating magnetic field
EPR effect	Enhanced permeability and retention effect
LPS	Lipopolysaccharide
MHT	Magnetic hyperthermia
MTB	Magnetotactic bacteria
MS	Magnetic session, time during which an AMF is applied on NP
NP	Nanoparticles
M-PLL	Iron oxide magnetosome minerals coated with poly-L-lysine
NP	Nanoparticles
NP backbone	Nanoparticle without any drug attached to it
GBM	Glioblastoma multiform
PNN	Poly nuclear neutrophils
